# Multiprofessional COPD care in Austria–challenges and approaches: Results of a qualitative study

**DOI:** 10.1007/s00508-018-1346-8

**Published:** 2018-05-28

**Authors:** Firuzan Sari Kundt, Nina Enthaler, Anna Maria Dieplinger, Michael Studnicka, Anna Knoll, Jürgen Osterbrink, Tim Johansson, Maria Flamm

**Affiliations:** 1 Institute of Nursing Science and Practice, Paracelsus Medical University, Strubergasse 21, 5020 Salzburg, Austria; 2 Institute of General, Family and Preventive Medicine, Paracelsus Medical University, Salzburg, Austria; 3 Department of Pneumology, Salzburger Landeskliniken, Paracelsus Medical University, Salzburg, Austria

**Keywords:** Respiratory nurse, Ambulatory care, Rehabilitation, Telemedicine, Patient education

## Abstract

**Background:**

Chronic obstructive pulmonary disease (COPD) is a frequent disease of the lungs. Its prevalence was estimated to be 26% in the Global Initiative for Chronic Obstructive Lung Disease (GOLD) I and 11% for GOLD II–IV in Austria. Globally, it ranks third in mortality rate. The particular challenge is that care for these patients falls short due to the lack of structured integrated care. The aim was to assess the current status of multiprofessional COPD care in Austria and identify gaps and potentials.

**Methods:**

We conducted guided focus group interviews between March and July 2016 addressing current COPD care and treatment gaps with the following professional and interest groups: general practitioners, nurses, patients, pharmacists, physiotherapists and pulmonologists. We interviewed 23 patients and 27 healthcare professionals. The interviews were transcribed verbatim and coded into 12 relevant categories.

**Results:**

There needs to be a shift in thinking from treatment-based care to prevention. Patients, just like healthcare professionals, need periodic updates and comprehensive information on this disease. Creating internet platforms with useful information for COPD patients and solving the data privacy issues of the Austrian electronic medical record (ELGA) are also perceived as viable steps. There is a need and request for healthcare professionals to work as a team with clear COPD management guidelines in the outpatient sector, the establishment of outpatient rehabilitation centers as well as creating a new professional profile, the COPD nurse.

**Conclusion:**

Current COPD care needs to be reorganized, particularly in the outpatient sector, to address the needs of patients and healthcare professionals.

## Background

Chronic obstructive pulmonary disease (COPD) is a common chronic disease, with prevalence rates estimated to be between 4% and 21% [1]. Globally, COPD has increased to become the third leading cause of death [2]. The largest risk factors for the development of COPD are exposure to active and passive smoking, genetic predisposition and occupational exposure to hazardous inhalants and small particles [3]. For both, men and women, the COPD prevalence rises steeply after age 40, exceeding 50% for the over 70 age group. Although these numbers are concerning, it is more disturbing that half of all COPD stage II+ sufferers do not receive a formal diagnosis, and hence, no adequate treatment [4]. Taking age, gender and urban-rural discrepancy into consideration, the COPD prevalence rate in Austria is estimated to reach 36% in 2020 [5]. Austrian COPD hospitalization rates (i. e. 310/100,000 population) are the second highest among the 20 compared European Union member states [6]. Studies have shown high mortality rates during hospitalization as well as after hospital discharge: the 90-day mortality is 10% with over half of these deaths happening shortly after hospital discharge [7]. To date in Austria, there is no comprehensive care network for the management of COPD and high rehospitalization rates show large gaps in outpatient care, rehabilitation and patient education [8]. Therefore, the Austrian healthcare system offers opportunities for improvement, such as decreased care gaps between hospital and outpatient care, increased networking between care sectors and refined outcome evaluations [9]. The Austrian healthcare system has been rated as a *low primary care system* [10] and has not yet implemented a structured integrated cooperation between different healthcare professionals. This fact complicates the provision of a structured and continuous care warranted by general practitioners in primary care [11].

In the international picture, various programs to counteract some of these issues have shown great successes in decreasing hospitalization and rehospitalization rates [12], the frequency of exacerbations as well as mortality all the while reducing treatment costs [13–18]. In addition, personalized action plans were able to reduce emergency department visits, hospital admissions and length of stay among participating patients by 60% [14], improve risk-adjusted patient outcomes, promote patient safety, increase patient satisfaction and optimize the use of resources [19].

### Aim

The objective of this qualitative study was to assess the current status of COPD care in Austria. The results of the focus group interviews were intended to generate examples for the development and implementation of a structured, integrated multiprofessional COPD care in Austria and with that increase patients’ health status, quality of life and self-care competencies. The research questions were:

1. Are there any potential improvement opportunities in integrated and multiprofessional COPD care concerning the care of patients and their caregivers as well as inpatient and outpatient care protocols?
2. Which viable improvement possibilities are available?

## Material and methods

### Design, participants and setting

The pre-study interdisciplinary structured intersectoral (ISI) COPD was an exploratory qualitative research effort to identify main and common factors of improvement in COPD care management in Austria. Therefore, we conducted focus group interviews with one group interview session for each professional, e. g. general practitioners (GP), pulmonologists, pharmacists, nurses and physiotherapists, and interest group (COPD support group). Among the professional groups, an expert interview approach was deemed as the most useful option to gather exploratory information on COPD care. Patients and their direct caregivers, on the other hand, are experiential experts who can provide valuable information on their individual needs.

### Recruitment

Inclusion criteria for participation were defined a priori. In order to participate, patients had to be over 18 years of age and have a formal COPD diagnosis. Patients with other pulmonary diseases were excluded. For the healthcare professionals, the inclusion criterion was to be directly involved in the care of COPD patients, preferably with some years of experience in caring for this patient group. Participant sampling was done using non-random sampling techniques based on availability and the snowball method. General practitioners were contacted by mail and then followed-up by telephone to recruit for participation. The pharmacists were recruited at the 49th annual science continuing education congress of the Austrian pharmacists’ association via information leaflets at the main entrance and an invitation on the information screens during the congress. Nurses and physiotherapists working with inpatients and outpatients, were recruited via email directed at all nursing and healthcare management offices of the Salzburg clinics. The email list with these addresses was obtained from the Austrian Society of Pneumology. In addition, we also recruited physiotherapists and pulmonologists from the outpatient sector via email and telephone calls. Due to unforeseen problems in the recruitment of pulmonologists from the Salzburg area, recruitment of this professional group was expanded to all of Austria and the interview mode was changed to individual interviews instead of focus groups. To recruit the patients, we contacted the local COPD support group and upon request, we were granted permission to hold the focus group interview on-site.

### Group interviews and informed consent

The focus group interviews were conducted by two trained project staff members from March through June 2016. The guided interview questions were gathered from the literature and were also the result of a kick-off meeting held with all professional healthcare groups and patients. The interview guide (Appendix A) was constructed using semi-structured open-ended questions. The interviews were held in strict adherence to the interview guide to ensure comparability and one of the project staff wrote a field diary. The interviews themselves were recorded by two digital audio recorders and then transcribed verbatim. Each participant gave informed consent before being admitted to formally participate in the interview and all participation was voluntary. Upon seeking ethics approval, the research team was informed that there is no need for an official ethics approval for this type of study as outlined in § 30 of the Salzburg Hospitals Act (SKAG) [20].

### Data analysis

The data were analyzed using qualitative content analysis techniques [21, 22]. Once the data was transcribed, we used MAXQDA 12© (Verbi GmbH, Berlin, Germany) to code the transcripts into 12 different categories (Appendix B) that were anchored on the questions from the interview guide and then paraphrased them. Each focus group or single interview was analyzed separately and later combined to assess common ground. Once the paraphrasing was finished, we created and defined new dimensions based on the clustered paraphrases to address the research questions [23]. To answer the research questions, we focused on two main dimensions: “current COPD care” and “potential solutions to current care issues.” These two dimensions yielded the following three subcategories: “educational measures,” “eHealth” and “structured integrated multiprofessional care” (Appendix C depicts the main themes of the potential solutions category). The three subcategories are expanded on in the following. The quotes used in this article were translated from German to English and back by two different native speakers. Both translators’ translations were congruent.

## Results

A total of 27 healthcare professionals from the medical sector (6 GPs, 7 pharmacists, 4 nurses, 6 physiotherapists and 4 pulmonologists; 15 women, 12 men) and 23 COPD patients (10 women, 13 men) were interviewed. Due to the large number of participants in the patient group, we had to split the group in two to make the interview manageable. Both interviews were conducted concurrently in separate rooms. Each interview lasted between 30 and 105 min. We received age and sex information on all participants, except for one patient who refused to disclose his age. The participants were between the ages 21 and 80 years, with a mean age of 57.2 years (Appendix D shows age and sex distribution of the participants).

### Educational measures

Both care providers and patients agreed that the issue of COPD has not yet achieved key illness status in society. Although COPD disorders are increasingly common in Austria, prevention and disease education are largely underrepresented and a lack of COPD awareness is being criticized.

Whereas some patients reported the fact that despite a diagnosis, their lack of knowledge about their own disease has continued for a long time, other patients described that when they received the diagnosis, they felt overwhelmed by the situation and were unable to follow and comprehend the information.

“When you get the diagnosis and have never heard of COPD, you’re a bit confused and you cannot follow it [the information], even though it is being explained thoroughly.” (Support group participant)

Responsibilities over who should conduct patient education consultations ranged from the physicians, specialists and nurses who suggested the professional field of COPD nurses already established abroad, extensions to the field of physiotherapy up to the increased involvement of pharmacists.

Patients need comprehensive patient education, similar to the one received by cancer patients. The physiotherapists, nurses and inpatient pulmonologists agreed and added that patients not only need one, but many follow-up consultations due to the progressively deteriorating nature of this disease. Physiotherapists specifically stated that the information for patients should contain comprehensive information on pathomechanisms of the disease, pharmaceutical treatment options as well as social, psychological, physiological and nutritional support options. This would help keep patient expectations realistic and potentially delay exacerbations.

Health education, and in particular, the correct handling of the devices, such as the inhalers, is also a major issue in successfully handling the disease.

“The patient, who knows (...) the [proper] handling of his respiratory device, [such as] the cleaning. (...) One really notices (...) in medical surgery that the gaps [between hospitalizations] (...) are much, much better [longer] as compared to the ones who receive the instruction, but who are totally overwhelmed [with this information] and have no appropriate support [system].” (Nurse)

General practitioners also expressed their desire to regularly receive the most current knowledge about COPD and evolving developments of its therapy.

“And you cannot (…) often enough (…) train physicians in this respect. I don’t think I am the only one, who time and time again, falls under the (…) required knowledge level automatically.” (General Practitioner)

In terms of prevention, although only one patient group mentioned this as important, all healthcare professional interviewees thought it critical for the patients to receive preventative health education.

“(…) prevention is, for sure, one of the most important things, yes.” (Nurse)

“And a difficulty in this context is of course that prevention is not satisfied in any way (…) we have a reparatory medical system. This means, first there is the damage, then it is repaired, but the avoidance [of damage] can hardly be found in the roots of social insurance.” (Inpatient pulmonologist)

All participants agreed on the lack of appropriate patient education and the need for clarification, preventative measures (e. g. in the form of television clips, videos, apps) and regular training for both COPD patients and their caregivers. There is consensus that COPD prevention in Austria is insufficient as compared to other countries, because the focus is placed on treatment rather than prevention of disease. According to focus group participants, prevention should start in kindergarten, but at the latest, following the Scandinavian model, in school, in order to be effective at all.

### EHealth solutions

A newly developed and recently adopted electronic medical record (EMR) system, ELGA, is trying to fill the gap in networking between the different healthcare providers and the patients. So far, this tool has been met with scepticism for reasons of data privacy, which is also the reason why many providers have opted out of it. However, an EMR system is an efficient way to connect all providers and track the patients’ medical history. Among the patient interviewees, there was widespread support for this new EMR resource.

“I am a supporter of ELGA. And I feel if ELGA really works and the people quit being so suspicious, the problem would be off the table.” (Support group participant)

This openness for an EMR without being intimidated by data privacy issues was prevalent among one patient group, the physiotherapists, nurses and one outpatient pulmonologist. All other interview groups were rather ambivalent on the idea of an electronic patient database.

In addition to ELGA, there currently is no local operational platform combining information on specifically trained local respiratory physiotherapists, appropriate sports groups or efficient smoking cessation offers to refer COPD patients to get additional assistance. The majority of patients are still not very likely to reach out for information and therapy options on their illness via digital media. Only one of the patient groups, the general practitioners, nurses and two of the pulmonologists were in favour of the idea of the utility of technical aids and contact points (e. g. WhatsApp), especially for patients who have recently been discharged from hospital.

“That they are coping well through these first days (...) at home. (...) That we call them (...) and they have a contact point. (...) Well, such technical aids we could also very well use. (...) Applications, which one can now simply download.” (Nurse)

Although both options, an EMR system (e. g. ELGA) and an online platform with information about recreational and therapy offers for COPD patients, is perceived as a good idea in theory but widespread support for the creation of these tools is still lacking. This attitude is partly an ethical issue (i. e. patient data privacy), but also stems from a lack of resources, such as time, money or internet affinity.

### Structured intersectoral care

#### Networking

The healthcare providers clearly distinguished between the inpatient area, where an uncomplicated and well-regulated multiprofessional exchange takes place, and the outpatient sector, where there is very little structured cooperation or exchange with other disciplines. The interviewees who are working with a multiprofessional team of specialists all agreed that good cooperation is the prerequisite for effective COPD therapy.

“They [hospitals] accept everyone who (...) comes in even without a referral, (…) completely without any networking and communication (…) and often I don’t find out about it until a year later that the patient exacerbated three times already.” (General Practitioner)

Although the patients themselves have varying opinions on how well the different providers cooperate, everyone agreed on the importance of a functional network of healthcare professionals. A professional that is often referred to as a feasible link in multiprofessional collaboration is the pharmacist. The healthcare providers as well as the patients can envision an increased involvement of this professional group. The pharmacists recognize their low-threshold access to the patients as a great potential and are willing to provide the patients with specific medication and device training, tests and measurements of vital functions.

“And I can certainly imagine that specially trained pharmacists could take on more responsibilities with the customers and check out together with them (…) how to apply my medication, where he [the pharmacist] specifically demonstrates how he [the patient] should do it. I believe that particularly the pharmacists (…) could assume a larger role, also because they are closer to the customer [patient] and they are there, easily reachable.” (Pharmacist)

Networking is perceived to be an ultimate necessity for the medical professions, including the pharmacists, to be able to offer the patients comprehensive care and avoid redundant therapeutic measures.

#### Disease management programs

Most interviewees agreed that the absence of structured COPD patient management plans, such as disease management programs (DMP), ultimately hurts the patients. Physiotherapists, nurses and general practitioners distinguished between the inpatient and outpatient sector, with the problem concentrating in the outpatient sector. Healthcare professionals in the outpatient sector dealing with COPD patients have to come up with their own particular disease management. The patients stated that the absence of structured and comprehensive COPD management programs subsequently leads to the patients being left without coordination and support, particularly immediately after hospital discharge.

“You are discharged from the hospital with wishes for recovery, with the documents and that’s it, more or less. There is no follow-up care, there are no therapies, such as physiotherapy, respiratory physiotherapy, nothing is offered.” (Support group participant)

It is strongly encouraged to implement guidelines by means of incorporating multiprofessional COPD care. The majority of the interviewees agreed with the establishment of a meaningful bridging solution after hospital discharge, which could contribute to a reduction in the number of exacerbations, and thus, lower rehospitalizations.

“A [low] rehospitalization can only take place if we invest in the sick person outside the hospital.” (Support group participant)

The establishment of outpatient COPD care guidelines is a key issue mentioned by all interviewees. These guidelines should cover the patients the minute they are discharged from the hospital to ensure continuous quality in care.

#### Outpatient rehabilitation

Most interviewees (i. e., pulmonologists, nurses and physiotherapists) appreciated the idea of a transitional area or period into the home environment or a post-hospital follow-up procedure to address some of the urgent care problems that surface right after hospital discharge. The establishment of a combined COPD ambulatory care management and outpatient rehabilitation center where multiprofessional care is provided could also help effectively deal with the gap in service. Outpatient rehabilitation facilities, which are already a standard in some cities, are not yet widely available. They provide necessary services to ensure continuity of care until the patients are able to cope on their own again. This would pose a sensible measure to relieve the inpatient area and adequately provide for the patient.

“It is necessary to understand that insufficient treatment ultimately results in significantly higher costs besides the individual suffering of the patients, which could be an incentive to make improvements.” (Resident pulmonologist)

The patients, on the other hand, are not entirely sold on the idea of an outpatient rehabilitation care center. Their worry concerns the extra costs associated with this service, but they also perceive it to be demanding and exhausting, not a place to recuperate.

“[An outpatient rehabilitation center is] not a hospital, but a rehabilitation and that is strenuous.” (Support group participant)

The establishment of an outpatient rehabilitation center is an idea that was perceived well by most interviewees; however, it would take a little persuasion work for the patients to also buy into it.

#### Respiratory/COPD nurse

Healthcare providers agreed that chronically ill patients need a stable accompanying factor helping them go through the various health stops and increase patient compliance while decreasing rehospitalization rates. A promising approach is the implementation of a respiratory or COPD nurse, a specifically trained nurse focusing on COPD. The healthcare professionals emphasize the importance of specifically trained nurses due to complex needs and comorbidities of COPD patients.

“If the patients were taken better care of at home, by the COPD nurse coming to your home, then perhaps some exacerbations could be easily managed at home and would not need to be taken to the hospital; but the patient alone cannot cope by him-/herself, s/he needs help. For this we have nothing, in Austria, we have quite a few acute beds, but relatively few other facilities, where these patients could be supported. So, I think home care is a good idea.” (Resident pulmonologist)

The patients, on the other hand, were somewhat opposed to the establishment of a respiratory nurse as interim care before and after a hospital stay. They doubted that this new type of professional would possess sufficient qualifications to be of any value to them.

“I personally don’t believe that such a nurse (…) that that would achieve anything.” (Support group participant)

On the topic of the respiratory/COPD nurse, the opinions diverged. The healthcare professionals thought it would be a useful addition to the care of COPD patients, whereas the patients took on a more careful stance.

#### Other resources

An additional issue is exhibited by the lack of outpatient respiratory physiotherapists in Salzburg. Most of the available outpatient respiratory physiotherapists are neither fully covered by the medical insurance nor is it possible to get an appointment within a reasonable amount of time (a few months).

“Well, the only thing I really want is an affordable respiratory physiotherapy in place.” (Support group participant)

The medical care of COPD patients is not the only factor in their well-being, but regular physical activity offers specifically tailored to the COPD patient are important measures as well. Information on these offers should be made easily accessible to all searching for them. Most patients agree in taking a proactive role in their own therapy utilizing a progressive self-help and competence building attitude.

“Through my physical activity every day, it has always been a bit better for me in recent years. And when I cannot be physically active due to an exacerbation, then I notice it is getting worse. And the more sports I do, the better I feel and do.” (Support group participant)

In addition, some interviewees agreed that families, relatives and even friends, play an important role in the well-being of the patient.

“Involving family members. In my opinion, they also belong to this interdisciplinary management.” (Physiotherapist)

There is a general consensus among the patients about taking charge of their own lives, be it with physical activity, social gatherings or seeking a respiratory physiotherapist. In our study, patients, who were the most physically and socially active, regardless of GOLD stage, also perceived their own health to be better than other patients, who were less proactive. The pulmonologists and physiotherapists agreed with the patients and supported the notion to increase and ease access to therapeutic resources for the patients. Physiotherapists, nurses and the pulmonologists thought that relatives should certainly be involved in patient care, whereas some patients responded more reservedly about involving their friends and relatives in their disease so as not to additionally burden them.

## Discussion

This study was the first of its kind taking a qualitative approach to get an overall picture on the shortcomings of COPD care in Austria giving voice to everyone involved in the care of COPD patients, including the patients themselves, since they are the utmost sufferers of these shortcomings. It provides a list of issues the patients and healthcare providers battle on a daily basis, but also potential solutions with a unique insight into the group perspectives on how to solve these issues.

Current COPD care in Austria is distinctly hospital-centered. It is a slowly progressing incurable disease with patchy care in the outpatient sector. Patients are released from the hospital to fend on their own, which usually leads them back into the hospital due to frequent exacerbations. There are no clear cut guidelines for the management after discharge for healthcare professionals to follow. Hence, the high Austrian rehospitalization rates are not surprising. If the providers act within a network of healthcare professionals, it is because they have personally built one for themselves. The absence of such networks was a common theme throughout all interviews, namely the lack of formal structures that facilitate interdisciplinary communication, and hence, build a functional network of care providers. These shortcomings place a large burden on all involved, but particularly on the patients, resources and the medical economy.

International evidence has successfully solved some of these issues by implementing various measures to lower hospitalization and rehospitalization rates, exacerbation frequencies and mortality and at the same time reducing treatment costs [13–18]. Special and targeted training of healthcare personnel, e. g. nurses, was not only able to effectively decrease COPD-related hospitalizations and associated care costs in Finland [15], but also empowered the patients in their self-care competencies, improve integrated care offers and successfully conduct prevention, case management and discharge programs [24]. In addition, personalized action plans and care pathways/care bundles were able to reduce emergency department visits, hospital admissions and length of stay among participating patients by 60% [14], improve risk-adjusted patient outcomes, promote patient safety, increase patient satisfaction and optimize the use of resources [19].

In the case of Austria, although the patients’ healthcare in the inpatient area is covered, there was a unanimous request of all interviewees to improve integrated care and extend the current coverage to include effective therapeutic services and products in the outpatient sector. The interviewees were well aware of the flaws of the current healthcare system and they were motivated to support potential and practical improvement efforts, however, they failed to carry it through due to financial and time shortcomings, but also in light of the question on how to realistically and effectively coordinate healthcare professionals in the management of COPD to ensure a working integrated multiprofessional care system. This culture of change is ongoing in many European countries and the trend to offer better care beyond the hospital is increasingly being asserted [25].

There already are different educational initiatives for patients and health care professionals (print or electronic media). Repeated patient education efforts to update and strengthen disease-related knowledge, medication training courses and smoking cessation assistance have proven to be efficient [26]; however, based on our results, these efforts seem to insufficiently reach the patients and providers.

The importance of e‑Health (internet platforms, apps) solutions was acknowledged by all interviewees. Overall, research in this respect has found positive effects of e‑Health on disease coping mechanisms [27–33]. In our study, the plethora of practical and informative offers on the internet was overall perceived positively, but not within the reach of every patient. The implementation of an overarching EMR system, such as ELGA, with full participation of all providers was strongly suggested by some of the interview groups (one patient group, one of the outpatient pulmonologists, the nurses and the physiotherapists), under the condition of solving the data privacy problem.

Adaptations to the ever-changing requirements of the healthcare system and healthcare needs are important steps. The establishment and improvement of an interdisciplinary and multiprofessional integrated care system are reasonable and should be implemented in a step by step fashion. The participants’ proposal for implementing outpatient rehabilitation facilities, where representatives of all relevant medical specialties work as a team, could be one such necessary step to solving current care issues.

There were quite a few suggestions about the necessity of structural changes. For one, a better network of care, in other words, interdisciplinary and also multiprofessional networks and enhanced communication flow are thought to improve healthcare outcomes. For the other, sufficient resource provision is crucial to cope with the complexity of comprehensive COPD care. Structured multiprofessional approaches and physical activity have shown to improve patients’ health outcomes and well-being [15, 34] as well as increase patients’ perception about feeling stronger, more empowered, supported and safer [29–31]. An already intact structural entity, the patient support group, has been demonstrated to be helpful in supporting fellow patients and disseminate updated information on the disease.

## Conclusion

In summary, we propose that the current care of COPD patients in Austria, in particular integrated care and multiprofessional cooperation within the outpatient sector, needs to be reconsidered. Austria’s high hospitalization and rehospitalization rate of COPD patients [6] is a clear plea for structural change. Improving structured COPD care could not only improve patients’ health status, but also promote autonomy, self-responsibility and self-empowerment in the patients’ daily life.

## Limitations

While planning the qualitative study, we intended to conduct focus group interviews with all different professional and interest groups; however, due to unforeseeable difficulties in recruiting a sufficient group of pulmonologists, we decided to conduct four individual/personal interviews, two hospital pulmonologists and two outpatient practicing pulmonologists. Furthermore, it is recommended to conduct focus group interviews with a number of participants ranging between five and eight [35]. Due to the mentioned recruitment issues, the focus group for the nurses had only four participants. On a positive note, participants of all focus groups represented a very heterogeneous group (in terms of age, gender and years of experience) enabling us to obtain diverse information. In this section, it should also be noted that all patients were participants of the support group, meaning they were physically fit enough to participate in a support group meeting, leaving all other patients who are not able to attend support group meetings, out of our circle of interviewees. A final limitation is that due to limited resources, most of our participants (i. e. general practitioners, patients, nurses and physiotherapists) were from Salzburg, whereas the pulmonologists and pharmacists were from all over Austria.

## Appendix A

### Interview guide for healthcare providers and patients

#### Guide for healthcare providers

Patient care

1. Please think of your COPD patients. How do you experience the typical COPD patient, how do you experience their relatives?
2. What kind of struggles do your COPD patients have?
3. Do you have enough resources for your COPD patients and can you fully use them?
4. What is your (personal) COPD patient management plan?
5. How do you deal with an acute exacerbation?
6. Do you have funds/opportunities for further education and training regarding respiratory diseases?

Networking

7. Which interdisciplinary interfaces exist in the care of COPD?
  - Is there cross-sectoral cooperation in this care?
8. How is the liaison between the inpatient and outpatient sector?
  - How is the process of care in the inpatient vs. the outpatient sector?
  - What is the biggest challenge in the care of COPD patients after hospital discharge?
  - What additional resources does the COPD patient need after a hospital discharge?
9. In your opinion, how are the patients and their relatives integrated into the therapy?
10. In your opinion, which job profiles are missing in the care of COPD patients?
  - Which professional profiles should be involved more in the care of COPD patients

Communication

11. How is the knowledge of patients and their relatives about their illness?

Potential solutions

12. What measures are needed to ensure a functional cross-sectoral integrated care system?
13. If you think of innovative international COPD care concepts, which projects would be good to implement here in Austria?
14. In your opinion, how can the rehospitalization rate be reduced?
15. What could realistically be covered by the medical insurance that is currently not covered and represents a massive barrier due to cost?
16. What role does prevention play in COPD care in Austria?

### Guide for patients

#### Patient care

1. In your opinion, how do you experience the typical COPD patient, how do you experience their relatives?
2. What are your difficulties as a COPD patient?
3. Do you have enough resources and can you fully use them?
4. What is your (personal) COPD patient management plan?
5. How do you deal with an acute exacerbation?
6. Do you have funds/opportunities for further education and training regarding respiratory diseases?

#### Networking

7. Which interdisciplinary interfaces exist in the care of COPD?
  - Is there cross-sectoral cooperation in this care?
8. How is the liaison between the inpatient and outpatient sector?
  - How is the process of care in the inpatient vs. the outpatient sector?
  - What is the biggest challenge in the care of COPD patients after hospital discharge?
  - What additional resources does the COPD patient need after a hospital discharge?
9. In your opinion, how are the patients and their relatives integrated into the therapy?
10. What professional profiles should be involved more in the care of COPD patients?
11. Whom would you contact as a COPD patient if you need therapy beyond regular care?

#### Communication

12. As a COPD patient, how would you rate your and your relative’s knowledge about your disease?

#### Potential solutions

13. What measures are needed to ensure a functional cross-sectoral integrated care system?
14. If you think of innovative international COPD care concepts, which projects would be good to implement here in Austria?
15. In your opinion, how can the re-hospitalization rate be reduced?
16. What could realistically be covered by the medical insurance that is currently not covered and represents a massive barrier due to cost?
17. What role does prevention play in COPD care in Austria?

## Appendix B

### Overview of the main concepts and subcategories for coding with MAXQDA

- Knowledge
  - Professionals
  - Patients
- Quality of life
  - Good
  - Bad
- Resources
  - Professionals
  - Patients
- Exacerbation
  - Professionals
  - Patients
- Inpatient stay
  - Professionals
  - Patients
- Care aspects (structure/interface/networking)
  - Professionals
  - Patients
- Costs
- Training
- Potential solutions (measures/innovative concepts/prevention)
- Smoking
- Addiction
- Significance (of the disease)

## Appendix C

### Table of potential solutions for all professional groups and patients

**Table:**

Potential Solutions		GP	PHA	PAT 1	PAT 2	PHYSIO	NUR	OP 1	OP 2	IP 1	IP 2
Educational measures	Health education and prevention measures	X	X	–	X	X	X	X	X	X	X
	Extensive health education talk	–	–	X	X	X	X	–	–	X	X
	Periodic trainings	X	X	X	–	X	X	X	X	X	X
E-Health	Online platform for training and therapy offers	X	–	–	X	–	X	–	X	X	–
	Introduction electronic medical record	–	–	X	–	–	–	X	–	X	–
	Electronic medical record with patient information	–	–	X	–	X	X	X	–	–	–
Structured multiprofessional integrated care	DMPs	X	–	–	–	–	X	–	–	X	–
	Guidelines	X	–	–	–	X	–	–	–	–	–
	COPD nurse	X	–	X	–	–	X	X	X	X	–
	Easy access to training and therapy offers	–	–	X	X	X	–	X	X	X	X
	Self-care competency	–	–	X	X	X	–	–	–	X	X
	Involve relatives	–	–	X	–	X	X	X	X	X	X
	Professional networking	X	X	X	X	X	X	X	X	X	X
	Pharmacy	X	X	X	–	–	–	–	–	–	–
	Specialized outpatient COPD care unit (rehabilitation, COPD outpatient care, interim nursing care)	–	–	–	X	X	X	–	X	X	–
	Outpatient rehabilitation for COPD patients	–	–	–	X	X	X	X	X	X	X

*GP* general practitioners, *PHA* pharmacists, *PAT* patient group, *PHYSIO* physiotherapists, *NUR* nurses, *OP* outpatient pulmonologist, *SP* inpatient pulmonologist, *DMP* Disease Management Program, the “X” means “mentioned by the particular group”

## Appendix D

### Table with participant age (years) and sex distribution

**Table:**

Group	Age	Sex
General practitioner	46	m
General practitioner	48	f
General practitioner	52	f
General practitioner	59	f
General practitioner	64	m
General practitioner	68	m
Nurse	25	f
Nurse	25	f
Nurse	34	m
Nurse	51	f
Patient in group 1	62	m
Patient in group 1	63	m
Patient in group 1	66	f
Patient in group 1	70	f
Patient in group 1	70	f
Patient in group 1	72	m
Patient in group 1	73	f
Patient in group 1	73	f
Patient in group 1	76	m
Patient in group 1	78	m
Patient in group 1	78	m
Patient in group 1	–	m
Patient in group 2	63	f
Patient in group 2	65	m
Patient in group 2	67	m
Patient in group 2	69	f
Patient in group 2	69	m
Patient in group 2	69	m
Patient in group 2	72	f
Patient in group 2	72	m
Patient in group 2	76	f
Patient in group 2	77	f
Patient in group 2	80	m
Pharmacist	41	f
Pharmacist	43	m
Pharmacist	50	f
Pharmacist	58	m
Pharmacist	59	m
Pharmacist	62	f
Pharmacist	70	m
Physiotherapist	21	f
Physiotherapist	22	f
Physiotherapist	23	f
Physiotherapist	24	f
Physiotherapist	39	m
Physiotherapist	44	f
Pulmonologist	39	m
Pulmonologist	45	f
Pulmonologist	64	m
Pulmonologist	65	m

*m* male, *f* female
